# The Effect of Transmuscular Quadratus Lumborum Block on Postoperative Opioid Consumption in Patients Undergoing Percutaneous Nephrolithotomy: A Randomized Controlled Study

**DOI:** 10.7759/cureus.18344

**Published:** 2021-09-28

**Authors:** Ugur Peksoz, Mine Celik, Haci Ahmet Alici, Suna Mehtap Celik, Ahmet Murat Yayik, Ali Ahiskalioglu

**Affiliations:** 1 Department of Anesthesiology and Reanimation, Ataturk University Faculty of Medicine, Erzurum, TUR; 2 Department of Algology, Medipol University School of Medicine, Istanbul, TUR; 3 Clinical Research, Development and Design Application and Research Center, Ataturk University Faculty of Medicine, Erzurum, TUR

**Keywords:** postoperative pain, nerve block, percutaneous nephrolithotomy, local anesthetic, interventional ultrasound

## Abstract

Background

This study aimed to investigate the effect of ultrasound-guided transmuscular quadratus lumborum block (QLB) on postoperative opioid consumption in patients undergoing percutaneous nephrolithotomy (PCNL).

Methodology

A total of 40 patients aged between 18 and 60 who were classified as American Society of Anesthesiologists status I-II and scheduled for unilateral PCNL were randomly divided into two groups. Patients in Group QLB (n = 20) received a single-shot QLB with 20 mL of 0.25% bupivacaine in the preoperative period. No intervention was performed in the control group (Group C, n = 20). Dermatomes affected by the block procedure were evaluated in the preoperative period in the group of patients who were administered the block procedure. General anesthesia was administered to all patients in both groups. In the postoperative period, opioid consumption, pain scores, side effects related to opioid consumption, and additional analgesic requirements were recorded.

Results

Opioid consumption was significantly lower in Group QLB compared to Group C at all times (p < 0.05). Postoperative visual analog scale (VAS) scores during the movement were significantly lower in Group QLB compared to Group C at all times (p < 0.05). VAS scores at rest were reported to be significantly lower in Group QLB compared to Group C, except for the eighth and twelfth hours (p < 0.05). The requirement for additional analgesic agents was significantly lower in Group QLB compared to Group C (p < 0.05).

Conclusions

QLB reduced postoperative opioid consumption and VAS scores by providing more effective analgesia compared to the control group in patients who underwent PCNL.

## Introduction

Renal lithiasis is an important disease affecting people’s health and daily life. This disease varies depending on factors such as age, sex, race, and geographical region. Although stones can be seen throughout the urinary system, they are most commonly seen in the kidney. Medical treatment, extracorporeal shock wave lithotripsy, retrograde intrarenal surgery, percutaneous nephrolithotomy (PCNL), and laparoscopic methods are currently used in treating kidney stones [1].

PCNL is an effective endoscopic method for treating renal staghorn stones, stones over 2 cm, or multiple stones. It is the gold standard in treating renal lithiasis because it is less invasive and has less morbidity compared to open stone surgery [2,3]. Although PCNL is a minimally invasive procedure, distension in the renal capsule and pelvicalyceal system, as well as the nephrostomy tube, can cause severe postoperative pain [4]. This postoperative pain is an important factor affecting morbidity related to pulmonary, cardiovascular, and emotional systems [5]. Appropriate and satisfactory treatment of pain reduces complication rates, duration of hospital stays, and patient costs [6].

Systemic opioids, nonsteroidal anti-inflammatory drugs (NSAIDs), and neuraxial methods such as epidural analgesia, paravertebral block, quadratus lumborum block (QLB), transversus abdominis plane (TAP) block, or local anesthetic infiltration along the nephrostomy tube can be employed to prevent postoperative pain caused by PCNL [5-11]. The use of NSAIDs is limited as these patients have potential renal problems. Opioids can cause important side effects such as respiratory depression, nausea-vomiting, sedation, and constipation. Regional anesthesia reduces the requirement for and consumption of postoperative opioids, protecting patients from such side effects [11]. Successful treatment of acute postoperative pain can prevent the development of chronic pain [12].

QLB was first introduced by Rafael Blanco as an alternative to the TAP block [13]. QLB has been employed in cesarean sections [13], abdominal surgeries [14-16], and hip joint surgeries [17] and provided effective postoperative analgesia. QLB can be performed in three ways under ultrasound (US) guidance. Transmuscular QLB was used in this study. During the procedure, local anesthetic fluid is applied between the quadratus lumborum muscle and psoas muscle fascia, with the fluid spreading along the anterior and middle thoracolumbar fascia [18,19]. Injection fluid has been demonstrated to spread from the thoracic paravertebral space to the retroperitoneal lumbar paravertebral region [20]. A previous study demonstrated fluid spreading to lumbar nerve roots in the psoas muscle [18]. Furthermore, spread to the ilioinguinal nerve, iliohypogastric nerve, T7 (67%), T8 (83%), and more often T9-T12 spinal nerve roots has been demonstrated [21].

In this study, our primary aim was to evaluate the effect of US-guided transmuscular QLB (QLB-III) on postoperative opioid consumption in patients scheduled to undergo PCNL. Our secondary aim was to evaluate the effect of US-guided transmuscular QLB on postoperative visual analog scale (VAS) pain scores.

## Materials and methods

Ethical approval for this randomized controlled study was provided by the local ethics committee. A total of 40 patients aged between 18 and 60 who were scheduled to undergo unilateral PCNL and were classified as American Society of Anesthesiologists status I-II during preanesthetic evaluation were included in this study. Written informed consent was obtained from all patients who agreed to participate.

The following patients were excluded from the study: (1) those with a history of heart disease, kidney disease, liver disease, hematological disease, peptic ulcer, gastrointestinal bleeding, central and peripheral neurological disease, and psychiatric disease; (2) drug allergy or allergy to amide-type local anesthetics; (3) those with definite contraindications in terms of regional anesthetic intervention (coagulopathy, bleeding diathesis, infection at the intervention site); (4) those with a history of narcotic and non-narcotic analgesic drug use within the 24-hour period preceding the operation, chronic pain history, drug and alcohol addiction, and a body mass index (BMI) of >35 kg/m^2^; and (5) patients who were not interested in participating in the study.

The patients were randomly allocated to two groups using Microsoft Office 365 Excel (Microsoft, Redmond, WA, USA): Group QLB (n = 20) and control group (Group C, n = 20). The patients were interviewed one day before the operation. They were informed about the procedure and the relevant process. The patients were informed about VAS measurement and the use of the patient-controlled analgesia (PCA) device.

**Figure 1:**
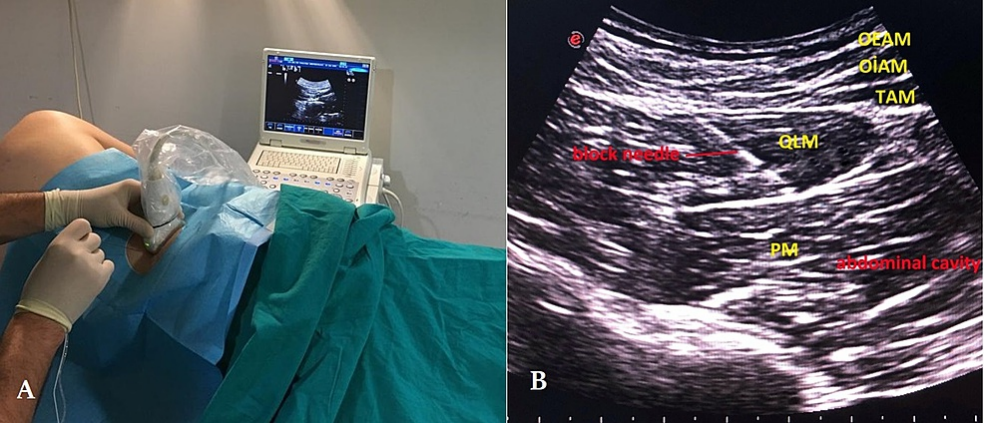
(A) Patient and ultrasound probe position for transmuscular QLB procedure. (B) Sonographic anatomy of the block. QLM: quadratus lumborum muscle; PM: psoas muscle; TAM: transversus abdominis muscle; OIAM: obliqus internus abdominis muscle; OEAM: obliqus externus abdominis muscle

Patients in Group QLB were taken to the regional anesthesia room 30 minutes before the procedure. After monitoring pulse, blood pressure, and SpO_2_, vascular access was established with a 22-G intravenous branule (Polyflon, India), and the block procedure was applied. Patients were placed in the right or left lateral decubitus position depending on whether the right or left side would be involved in the PCNL procedure (Figure 1A). The block area was cleaned with a surgical antiseptic solution. The curvilinear probe (Esaote MyLab30, CA631 high-frequency probe, UK) was sterilized using a surgical antiseptic solution and covered with a sterile sheet. The sterile probe was then transversely placed on the crista iliaca. Subsequently, the probe was cranially moved and oriented until the external oblique muscle, internal oblique muscle, and transversus abdominis muscle were clearly visualized. The US probe was posteriorly oriented. When the quadratus lumborum muscle was clearly visualized, the probe was held steady. The quadratus lumborum muscle, the latissimus dorsi muscle, and the transverse process of the vertebrae were visualized. A 20-G, 100-mm block needle (Stimuplex® Ultra 360®, Braun, Germany) was advanced posterolaterally to anteromedially using the “in-plane” technique. The quadratus lumborum muscle, psoas major muscle, and erector spina muscle were visualized. The needle was passed via the quadratus lumborum muscle transmuscularly and directed between the quadratus lumborum muscle and the psoas major muscles (Figure 1B). After confirming the injection site using 2 mL 0.9% NaCl, 20 mL 0.25% bupivacaine (Buvasin®, Vem Pharmaceuticals, Turkey) was injected between the fasciae of the two muscles. Local anesthetic distribution was observed, and a sensory examination was performed every 10 minutes using the hot-cold method. The block was considered to be successful in cases who did not feel cold in T10-L1 dermatomes at the end of 20 minutes, following which the case was included in the study. The dermatomes where the block was effective were recorded [18].

No procedure was performed for patients in Group C before the surgery, and they were taken to the operating room at the time of surgery.

Standard electrocardiogram, noninvasive blood pressure, and SpO_2_ monitoring were performed in both groups in the operating room. Vascular access was established with a 20-G intravenous (IV) cannula and a 6 mL/kg crystalloid infusion was started. Anesthesia was induced with IV 2 mg/kg propofol and muscle relaxation with IV 0.6 mg/kg rocuronium. For analgesic purposes, IV 2 µg/kg fentanyl was administered. Endotracheal intubation was performed after achieving satisfactory muscle relaxation. When necessary, 0.1 mg/kg rocuronium was administered during the operation for muscle relaxation. Anesthesia was maintained with 1−2% sevoflurane, 50% N_2_O, and 50% O_2_.

Age, sex, weight, height, BMI, duration of surgery and anesthesia, operative side (right or left), and stone characteristics were recorded for all cases included in the study.

All surgical procedures were performed by the same surgical team using the same surgical technique. The procedure was initiated by placing a ureteral catheter in the lithotomy position under the guidance of a 19-F rigid cystoscope. The patients were then placed in the prone position. Retrograde pyelography was performed by administering radiopaque material through the ureteral catheter. Percutaneous entry into the kidney was achieved using an 18-G needle and a guidewire. A 24-F sheath was placed after the access was opened with an Amplatz dilator. Stones were fragmented using a pneumatic lithotripter. In the postoperative period, a nephrostomy tube was placed in the presence of residual stones, active bleeding, and possible extravasation in the calyceal system. A 4.8-F double J ureteral stent was placed in patients who did not have a nephrostomy tube inserted.

Approximately 30 minutes before the end of the surgery, in both groups, 1,000 mg IV paracetamol was administered, and then repeated postoperatively every six hours. At the end of the surgery, no antiemetic agents were administered. Postoperative analgesia was provided with intravenous PCA using the same protocol in both groups. Postoperative follow-up and evaluation of patients were performed by a healthcare professional from the anesthesia team who was blinded to the study.

For postoperative analgesia, PCA was attached in the Post-Anesthesia Care Unit (PACU) for all patients postoperatively. The PCA device was prepared with fentanyl and programmed at a concentration of 10 µg/mL, loading dose of 50 µg, lockout time of 20 minutes, bolus dose of 25 µg, without basal infusion, and was continued for 24 hours. Patients with an Aldrete score of 9 or above were sent to the service. Rescue analgesia of 25 mg meperidine was administered to all patients with a VAS score of 4 and above in the recovery room and during follow-up. The need for rescue analgesia was recorded during the postoperative period.

Postoperative fentanyl consumption was recorded at 0-4 hours, 4-8 hours, 8-24 hours, and 24 hours. Side effects related to opioids such as nausea, vomiting, itching, constipation, and urinary retention were also recorded. First analgesic time (the time between block procedure and first-time pressing PCA), total PCA demand in 24 hours, total PCA administered in 24 hours were recorded.

VAS was used to evaluate postoperative pain. Postoperative pain levels were evaluated in the PACU and after one, two, four, eight, twelve, and twenty-four hours. Pain assessment was performed at rest (while the patient was lying down) and during movement (when the patient was in a sitting position).

After obtaining ethical committee approval, we conducted a preliminary power analysis. Seven patients were followed in each group. In our preliminary study for fentanyl consumption in both groups, the standard deviation was reported to be 131.76 in Group QLB and 128.26 in Group C. The difference in average 24-hour fentanyl consumption between the two groups was 172.5 µg. The number of patients required for each group was determined to be 14 using Russ Lenth’s Piface Java module with a power of 95% and an alpha error of 0.05. However, considering the possibility of patients being excluded from the study, it was decided to include 20 patients in each group.

SPSS version 20.0 (SPSS Inc., Armonk, NY, USA) was used for statistical analysis. Normal distribution of numerical data was assessed using Kolmogorov-Smirnov and histogram tests. Demographic data were assessed using Mann-Whitney U-test, independent samples test, and chi-square test, whereas operation-related data were assessed using Mann-Whitney U-test and independent samples test. Data on kidney stone characteristics were analyzed using Mann-Whitney U-test and chi-square test. Postoperative opioid consumption was analyzed using Mann-Whitney U-test, whereas data on the level and duration of opioid requirements were analyzed using Mann-Whitney U-test and independent samples test. Postoperative pain scores (VAS) at rest and during movement were analyzed using Mann-Whitney U-test and independent samples test. Data on side effects related to opioid consumption were analyzed using chi-square test. A p-value of <0.05 was considered statistically significant.

## Results

**Figure 2:**
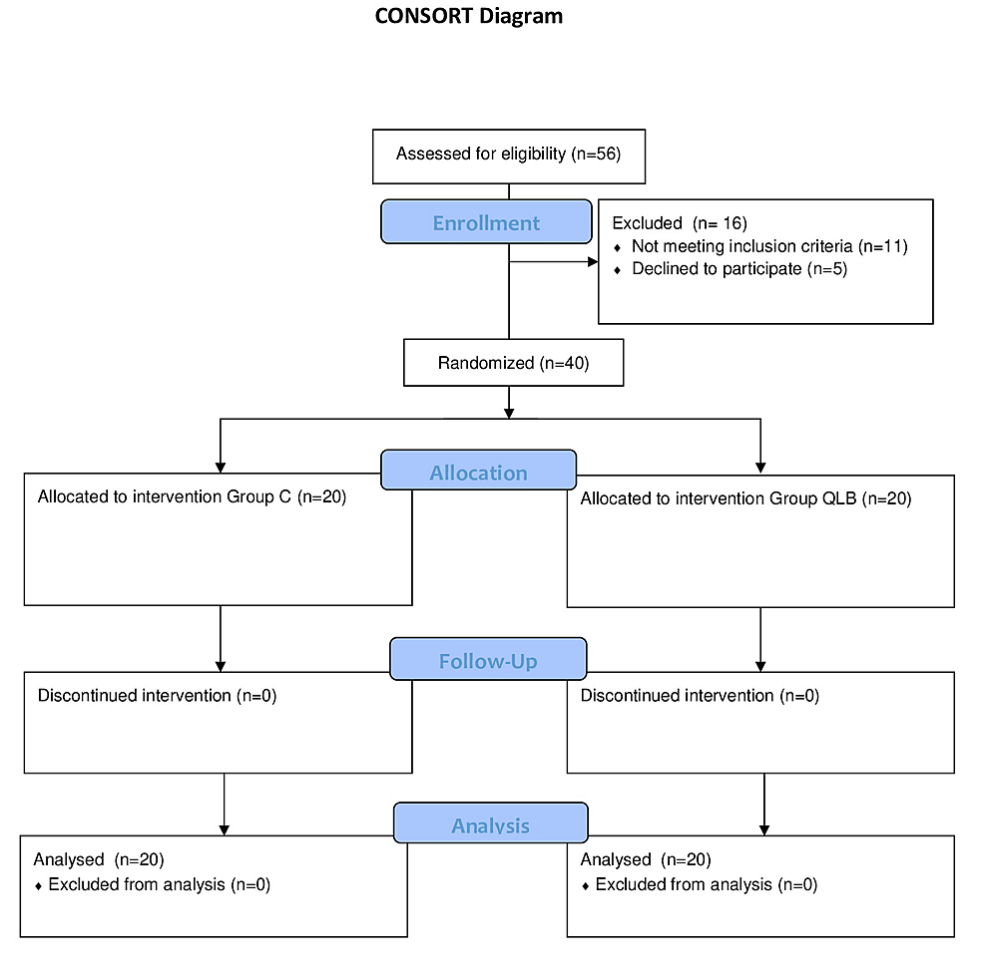
Consolidated Standards of Reporting Trials (CONSORT) flow diagram.

Eligible patients for the study were analyzed for the primary outcomes and are shown in the CONSORT flow diagram (Figure 2).

There was no statistically significant difference among the demographic data of the patients in terms of sex, age, weight, height, and BMI (p > 0.05). Additionally, no statistically significant difference was seen between the two groups in terms of the data related to the duration of surgery and the duration of anesthesia (p > 0.05) (Table 1).

**Table 1:** Demographic characteristics and operation data of study participants. Values are presented as number or mean ± standard deviation. ^a^ Mann–Whitney U-test; ^b^ Independent samples test; ^c^ Pearson’s chi-square test. QLB: quadratus lumborum block; BMI: body mass index

	Group C (n = 20)	Group QLB (n = 20)	P-value
Age (years)	49.10 ± 6,94	43.00 ± 12.80	0.213^a^
Weight (kg)	80.55 ± 9.84	76.10 ± 11.21	0.190^ b^
Height (cm)	170.20 ± 8.92	168.15 ± 8.48	0.461^ b^
BMI (kg/m^2^)	29.20 ± 2.65	27.70 ± 4.80	0.229^b^
Gender (Female/Male)	9/11	7/13	0.519^c^
Duration of anesthesia (minutes)	124.50 ± 40.55	134.75 ± 45.87	0.406^a^
Duration of surgery (minutes)	152.25 ± 37.78	163.50 ± 44.72	0.396^b^

The data on kidney stone characteristics including stone size, type of surgery (postoperative nephrostomy tube use), location of the stone, stone type, surgical target calyx, and stone size were compared. There was no statistically significant difference between the groups (p > 0.05).

Total postoperative fentanyl consumption at 0-4 hours, 4-8 hours, 8-24 hours, and 24 hours and total meperidine consumption for 24 hours were statistically significantly lower in Group QLB compared to Group C at all hours (p < 0.05) (Table 2). The total amount of PCA demand in 24 hours and the total amount of PCA administered in 24 hours were statistically significantly lower in Group QLB compared to Group C. The time of first PCA demand was statistically significantly longer and later in Group QLB than Group C. Furthermore, the analgesic demand of Group QLB emerged later (Table 3).

**Table 2:** Postoperative opioid consumption. Values are presented as number or mean ± standard deviation. ^a^ Mann–Whitney U-test. QLB: quadratus lumborum block

	Group C (n = 20)	Group QLB (n = 20)	P-value
0–4 hours (Fentanyl, µg)	180.00 ± 52.94	81.25 ± 41.26	<0.001^a^
4–8 hours (Fentanyl, µg)	212.50 ± 47.64	80.00 ± 62.09	<0.001^a^
8–24 hours (Fentanyl, µg)	270.00 ± 69.59	141.25 ± 104.90	<0.001^a^
Total 24 hours (Fentanyl, µg)	660 ± 120.14	302.50 ± 163.82	<0.001^a^
Total 24 hours (Meperidine, mg)	121.25 ± 31.70	51.25 ± 32.92	<0.001^a^

**Table 3:** Patient-controlled analgesia device data. Values are presented as number or mean ± standard deviation. ^a^ independent samples test; ^b^ Mann–Whitney U-test. QLB: quadratus lumborum block; PCA: patient-controlled analgesia

	Group C (n = 20)	Group QLB (n = 20)	P-value
PCA demand	29.05 ± 6.62	13.80 ± 7.98	<0.001^a^
PCA given	25.60 ± 4.99	10.65 ± 6.73	<0.001^a^
First analgesic time (minute)	159.75 ± 41.15	220.50 ± 44.42	<0.001^b^

Comparing VAS scores at rest, there was a statistically significant difference between Group QLB and Group C in the PACU at one, two, four, and twenty-four hours (p < 0.05), whereas there was no statistically significant difference between the two groups at reat measured at eight and twelve hours (p > 0.05). The VAS scores at rest in the PACU at one, two, four, and twenty-four hours were statistically significantly lower in Group QLB. Comparing VAS scores during movement, a statistically significant difference was seen between Group QLB and Group C in the PACU at one, two, four, eight, twelve, and twenty-four hours (p < 0.05). VAS scores during movement in the PACU at one, two, four, eight, twelve, and twenty-four hours were statistically significantly lower in Group QLB (Table 4).

**Table 4:** Postoperative pain scores. Values are presented as number or mean ± standard deviation. ^a^ Mann–Whitney U-test; ^b^ Independent samples test. QLB: quadratus lumborum block; VAS: visual analog scale; PACU: Post-Anesthesia Care Unit

	Group C (n = 20)	Group QLB (n = 20)	P-value
VAS at rest			
PACU	5.20 ± 2.14	1.65 ± 1.60	<0.001^a^
1 hour	4.90 ± 1.94	1.95 ± 1.32	<0.001^a^
2 hours	4.10 ± 1.59	2.55 ± 1.54	0.005^a^
4 hours	3.70 ± 1.13	2.40 ± 1.50	0.003^a^
8 hours	3.30 ± 0.92	2.70 ± 1.34	0.060^a^
12 hours	2.50 ± 0.60	1.95 ± 1.05	0.128^a^
24 hours	1.65 ± 0.58	1.10 ± 0.91	0.007^a^
VAS at movement			
PACU	7.10 ± 2.34	2.85 ± 2.01	<0.001^a^
1 hour	7.15 ± 2.35	3.25 ± 1.71	<0.001^a^
2 hours	6.35 ± 1.98	4.10 ± 2.20	0.002^b^
4 hours	6.05 ± 1.28	3.50 ± 2.01	<0.001^a^
8 hours	5.45 ± 1.23	4.40 ± 2.11	0.040^a^
12 hours	4.40 ± 1.57	3.15 ± 1.76	0.023^a^
24 hours	2.90 ± 1.11	2.15 ± 1.78	0.030^a^

No statistically significant difference was observed between the two groups in terms of side effects other than itching (p > 0.05). Itching was reported to be statistically significantly higher in Group C (p < 0.001) (Table 5).

**Table 5:** Side effects associated with opioid consumption. Values are presented as number. ^a^ Fisher’s exact test. QLB: quadratus lumborum block

	Group C (n = 20)	Group QLB (n = 20)	P-value
Nausea (yes/no)	8/12	3/17	0.077^a^
Vomiting (yes/no)	5/15	2/18	0.212^a^
Constipation (yes/no)	2/18	0/20	0.147^a^
Itching (yes/no)	9/11	0/20	<0.001^a ^
Urinary retention (yes/no)	1/19	0/20	0.311^a^

**Figure 3:**
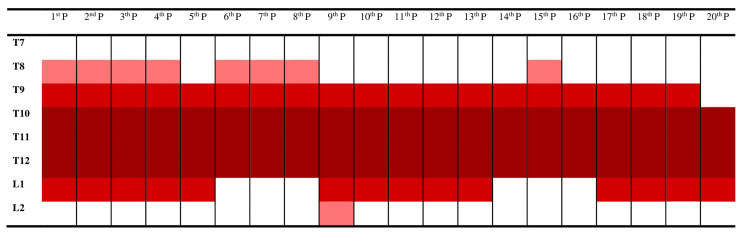
Dermatomes affected by QLB. Dark red: most commonly affected; red: more frequently affected; light red: less frequently affected. P: patient; T: thoracic; L: lumbar; QLB: quadratus lumborum block

Previous studies have shown that QLB is effective in T7-L1 dermatomes. In our study, the examinations we performed after the block procedure in Group QLB revealed that the upper limit of the block reached T8 in eight patients and T9 in eleven patients. In one patient, the level reached T10. The lower limit of the block reached T12 in six patients, L1 in thirteen patients, and L2 in one patient (Figure 3). All blocks were applied successfully.

## Discussion

The results of our study show that US-guided transmuscular QLB significantly reduced opioid consumption and postoperative pain scores.

Treatment methods for urinary system stone disease have changed over the years with technological development. PCNL, one of the common treatment methods used today, is a procedure with proven efficacy and reliability [2]. PCNL is a much more minimal procedure than open surgery and has multiple advantages. Improved quality of life and reduced hospital stay are the main advantages during the postoperative period. However, one of the most important challenges that arise during this period is pain which is yet to be tackled completely.

Postoperative pain, which occurs because of surgery and often terminates upon recovery of traumatized tissue, is a process that affects various organ or system functions. The circulatory system, gastrointestinal functions, autonomic nervous system, neuroendocrine system, and neuropsychiatric functions are the primary systems that are affected. If the complications that arise due to pain are treated incorrectly or inadequately, it prolongs the duration of hospital stay and reduces the quality of life, possibly resulting in chronic pain. Nausea, vomiting, sleep disorders, urinary retention, impaired bowel function, respiratory depression, thromboembolic and pulmonary complications, and circulatory failure are just a few of the adverse conditions that may develop. Pain management not only increases patient comfort but also enables early mobilization by improving all systems with changing physiological activity, especially the respiratory and gastrointestinal systems, and reduces postoperative morbidity and mortality. Furthermore, postoperative pain management not only ensures patient comfort but also allows early mobilization by preventing respiratory and gastrointestinal dysfunction and reducing mortality and morbidity [22].

Tissue damage is often observed in the surgical area during PCNL. In addition, renal capsule and renal parenchyma dilatation occur. In certain cases, a nephrostomy tube is placed. In the postoperative period, pain occurs because of the nociceptive stimuli that occur as a result of these factors [23].

Systemic opioids, NSAIDs, neuraxial methods such as epidural analgesia, paravertebral block, QLB, TAP block, or local anesthetic infiltration along the nephrostomy tube can be employed to prevent postoperative pain caused by PCNL [5-11].

QLB has been successfully applied in various groups of surgical patients for postoperative pain management [13-15,24]. The quadratus lumborum muscle runs along the posterior abdominal wall and is located lateral to the psoas major muscle. This muscle starts from the inner part of the posterior part of the iliac wing and four small tendons attach this muscle to the lower medial of the 12th rib and the transverse processes of the L1-L4 lumbar vertebra. The subcostal nerve, iliohypogastric nerve, and ilioinguinal nerve run between the quadratus lumborum muscle and the transversalis fascia. Lateral branches of the thoracoabdominal nerves that receive sensation lateral to the thorax from the iliac wing, abdomen, and upper femur arise near the angle of the rib. The subcostal and iliohypogastric nerve pass through the anterior surface of the quadratus lumborum muscle [18].

The injection fluid in QLB spread along the thoracolumbar fascia to the thoracic paravertebral space and the lumbar paravertebral region [20]. Spread to the ilioinguinal nerve, iliohypogastric nerve, T7 (67%), T8 (83%), and more often T9-T12 spinal nerve roots has been demonstrated [21]. A previous study has demonstrated the spread to lumbar nerve roots in the psoas muscle [25].

Few studies in the literature have reported the advantages of different variations of QLB over each other. Several studies have demonstrated that transmuscular QLB is more effective in the postoperative period than other types of QLB [26]. Therefore, transmuscular QLB was used in our study. Moreover, the QLB procedure can be performed under general anesthesia. In this study, the procedure was performed while patients were awake to ensure that the block occurred and to determine the dermatomes where the block was effective.

QLB has been used to prevent pain after the PCNL procedure. In a study, QLB-3 administered to patients at the end of the PCNL procedure reduced postoperative morphine consumption and pain levels while reducing postoperative mobilization time and duration of hospital stay [27]. In another study, QLB-1 performed at the end of the PCNL procedure significantly reduced postoperative pain and morphine consumption [28]. In another study, QLB-2 and transmuscular QLB were preoperatively administered to patients under general anesthesia. Compared to the control group, the amount of sufentanil consumption in the intraoperative period was lower in the patient groups where these two blocks were applied. The 24-hour VAS scores of the groups in which the block was applied were reported to be lower. In the postoperative period, muscle strength was weaker on the side where transmuscular QLB was applied [29]. Postoperative pain levels and morphine consumption were demonstrated to be lower in patients who underwent PCNL with transmuscular QLB after spinal anesthesia [9].

QLB is superior to these two blocks in terms of affecting both the paravertebral block area and the TAP block area [20]. Although paravertebral block provides good unilateral analgesia, it is associated with a high probability of pneumothorax, hypotension, and vascular injury [30]. However, epidural block applications appear to be more invasive than QLB because of diffuse hypotension, risk of intrathecal drug administration, intrathecal infection, epidural hematoma, or other neurological complications. Peritubal infiltration reduces postoperative pain and opioid consumption in patients undergoing PCNL. It is applied to more superficial tissues such as the skin, subcutaneous tissue, and renal capsule. However, a previous study demonstrated that paravertebral block reduced parietal and visceral pain better and was more effective than peritubal infiltration [11].

In our study, pain assessment was performed using the VAS scoring system on movement and at rest in the postoperative 24 hours. These assessments showed that the VAS scores at rest in the PACU and one, two, four, and twenty-four hours were statistically significantly lower in Group QLB than in Group C. Although no statistically significant difference was observed at eight and twelve hours, the VAS scores were numerically lower in Group QLB. The VAS scores during movement in all time periods were lower in Group QLB than in Group C.

Additionally, postoperative fentanyl consumption, meperidine consumption for rescue analgesia, PCA demand, and the number of PCA administered were lower in Group QLB than in Group C. Furthermore, the first analgesic time was later in Group QLB compared to Group C. Considering the side effects such as nausea, vomiting, constipation, itching, and urinary retention caused by opioid consumption, itching was reported to be statistically significantly lower in Group QLB than in Group C. Other side effects were numerically lower in Group QLB; however, no statistically significant difference was reported between the two groups.

According to the examinations performed after transmuscular QLB to determine the dermatomes where the block was effective, it was observed that the upper limit of the block reached T8 in eight patients and T9 in eleven patients. In one patient, this upper level reached T10. The lower limits of the block reached T12 in six patients, L1 in thirteen patients, and L2 in one patient. Based on these dermatome areas, it can be concluded that transmuscular QLB can be applied for different surgeries.

We attribute the lack of statistically significant difference in the at-rest VAS scores at eight and twelve hours to the limited number of patients included in the study. Although the average VAS scores were not statistically significant, the mathematical average was reported to be lower in Group QLB. The analgesic effect of bupivacaine, used as a local anesthetic, lasts approximately four to six hours. The low pain levels and reduced opioid consumption in Group QLB for approximately 24 hours may be associated with successful reduction of acute pain and a decrease in the release of pain mediators. The low pain levels of the patients in Group QLB in the acute period may have caused them to feel emotionally comfortable as well as lower pain level in the following hours.

Our study has some limitations. First, no information was available concerning the preoperative pain levels of patients. Preoperative pain levels can affect postoperative analgesic consumption. Second, the study was only single-blinded. No sham injection was administered to Group C, and therefore the placebo effect of injection could not be assessed. Third, the study sample size was determined based on opioid requirements, which was the primary aim of the study. Postoperative VAS scores and side effects due to opioid consumption may not be fully identified with small sample size. Further studies with larger sample sizes are needed. Fourth, the VAS score, which we used to evaluate the pain levels of patients, may not be an objective evaluation method. Patient responses to pain are extremely variable. Finally, patient satisfaction data were not recorded in the study, which is one of the important limitations of the study.

## Conclusions

US-guided transmuscular QLB procedure provided more effective analgesia compared to no block intervention in patients who underwent PCNL in the postoperative period. It reduced opioid consumption and decreased pain levels. Transmuscular QLB administration may be beneficial for patients undergoing PCNL.
